# Sociodemographic influences on private and professional contact behaviour during the COVID-19 pandemic in Germany: cross-sectional analysis based on a Regional Blood Donor Cohort

**DOI:** 10.1186/s13104-024-06867-9

**Published:** 2024-07-27

**Authors:** Robert Pohl, Christoph Stallmann, Pauline Marquardt, Ute Bank, Jacqueline Färber, Lotte Scheibler, Hans-Gert Heuft, Achim J. Kaasch, Christian Apfelbacher

**Affiliations:** 1 Institute of Social Medicine and Health Systems Research, Medical Faculty, Otto von Guericke University Magdeburg, Magdeburg, Germany; 2 Institute of Medical Microbiology and Hospital Hygiene, Medical Faculty, Otto von Guericke University Magdeburg, Magdeburg, Germany; 3 Institute for Transfusion Medicine and Immunohaematology, Medical Faculty, Otto von Guericke University Magdeburg, Magdeburg, Germany

**Keywords:** COVID-19, Contact reduction, Blood donors, SeMaCo study

## Abstract

**Objective:**

The COVID-19 pandemic has had significant health and socioeconomic impacts worldwide. Extensive measures, including contact restrictions, were implemented to control the spread of the virus. This study aims to examine the factors that influenced private and professional contact behaviour during the COVID-19 pandemic.

**Results:**

We used baseline data (January–April 2021) from the SeMaCo study (Serologische Untersuchungen bei Blutspendern des Großraums Magdeburg auf Antikörper gegen SARS-CoV-2), a longitudinal, regional cohort study assessing COVID-19 seroprevalence in blood donors from Magdeburg and surrounding areas in Germany. In the blood donor cohort (*n* = 2,195), there was a general reduction in private contacts (by 78.9%) and professional contacts (by 54.4%) after March 18, 2020. Individuals with higher education reduced both private (by 84.1%) and professional (by 70.1%) contacts more than those with lower education levels (private contacts 59.5%; professional contacts 37%). Younger age groups (18–30 years) reduced private contacts more frequently (by 85.4%) than older individuals (61–83 years, by 68.6%) and demonstrated a higher likelihood of private contact reduction compared to older age groups (51–60 years: odds ratio (OR) 0.45 [95% [CI] 0.32–0.65]; 61–83 years: OR 0.33 [95% [CI] 0.22–0.48]).

**Supplementary Information:**

The online version contains supplementary material available at 10.1186/s13104-024-06867-9.

## Introduction

The COVID-19 pandemic has caused considerable mortality and hospitalization rates globally [1–3]. In order to reduce these effects, a fundamental part of the initial non-pharmaceutical interventions (NPI) to control the spread of the SARS-CoV-2 virus in Germany was the restriction of social contacts. The association of NPI with a significant reduction in the spread of COVID-19 in Germany (and generally in Europe) [4] has been substantiated by various models [5, 6]. At the same time, there has been an urgent call for research on the impact of NPI on the protection of older adults during the early stages of the pandemic [7]. Especially at the beginning of the pandemic, there were no specific medical treatments (e.g. vaccinations) to curb the spread of the SARS-CoV-2 virus. This underscored the need to adhere to NPI such as social distancing and contact restrictions. Nevertheless, reducing the frequency of contact to other individuals is a significant factor influencing the spread of infectious diseases and is recommended in international guidelines for COVID-19 prevention [8].

On March 18, the World Health Organization (WHO) held a global media conference on COVID-19 to highlight the danger and the importance of physical distancing measures [9]. Despite the crucial role of reducing personal contacts after 18 March, 2020 in curbing the SARS-CoV-2 pandemic, there have been limited studies examining the socioeconomic factors influencing adherence to social distancing measures in Germany [10]. Waldhauer et al. (2022) have found that individuals with higher educational status tended to have lower private contact reductions compared to those with low and medium educational. Additionally, participants with lower educational and occupational status showed less professional contact reduction [11]. Building on these findings, the aim of our study was to explore how various socioeconomic characteristics relate to the reduction of private and professional contacts during the early phase of the COVID-19 pandemic in Germany, using data from a regional blood donor cohort.

## Method

This study is reported according to the STrengthening the Reporting of OBservational studies in Epidemiology (STROBE) checklist for cross-sectional studies [12, 13].

### Study design

We used baseline data (20 January, 2021, to 30 April, 2021) from the SeMaCo study, a prospective, longitudinal cohort study aimed at determining the seroprevalence of SARS-CoV-2 among blood donors at the University Hospital Magdeburg Blood Donation Service, Saxony-Anhalt, Germany. Over a period of 21 months, the SeMaCo study examined blood donors during four data collection phases, each lasting four months. In addition to serological examinations, participants in the SeMaCo study filled out questionnaires capturing socio-demographic characteristics, frequency of contacts, non-professional care-giving activities, and incidence of COVID-19 (Questionnaire1), as well as information on vaccinations and vaccine attitudes (Questionnaire2). The questionnaire data was recorded on the same day as the blood samples were taken. The survey of contact frequencies is based on a retrospective approach.

The entire methodological approach has been described in the study protocol [14]. Additionally, a characterization of the SeMaCo baseline cohort, including the variables used in Questionnaire 1 and Questionnaire 2, are published elsewhere [15].

In order to ensure high comparability, the majority of questions regarding contact behaviour in Q1 were based on the study protocol of the Robert Koch Institute’s (RKI) Corona Monitoring [16].

### Variables

#### Private and professional contacts

We collected variables that captured reductions in private and professional contacts of the Participants after 18 March, 2020. To avoid bias, participants who indicated in the retrospective questions from Questionnaire 1 that they had no contacts before 18 March, 2020 and thus could not reduce these contacts after that date were excluded from the analyses.

To capture changes in private and professional contacts after 18 March, 2020, participants were asked: “Have these direct contacts with friends, relatives, and neighbours changed since March 18, 2020?” and “Have these direct contacts with colleagues or co-workers changed since March 18, 2020?” The response options were “No change,” “Yes, fewer contacts,” and “Yes, more contacts”. Changes in direct contacts in the private environment (friends, relatives, neighbours) and in the professional environment (colleagues or co-workers) were dichotomously coded (“Yes, contacts have decreased” vs. “No change” and “Yes, they have increased”).

Educational status was assessed based on the Comparative Analyses of Social Mobility in Industrial Nations (CASMIN) classification [17]. In order to identify participants actively engaged in the labour market for inclusion in the analysis of professional contacts, information on employment status was used to create a new variable categorized as “Active on the labour market” and “Not active on the labour market” Participants who were not active on the labour market (e.g., retired, pensioneers, school pupil, and students) were excluded from the analysis of professional contacts.

In order to identify a SARS-CoV-2 infection, a variable representing unvaccinated seropositive blood donors was created. IgG antibodies were detected using the LIAISON^®^ SARS-CoV-2 TrimericS IgG Antibody Test by DiaSorin (using a cutoff of ≥ 33.8 BAU/ml as a positive result), and vaccination status was obtained from the quantitative surveys, along with all other variables (gender, age, education, marital status).

## Results

The sample presented here differed according to the reduction in contact in the private and professional sectors. Each participant had a SARS-CoV-2 test result and the opportunity to complete the two questionnaires. As a result, the number of cases for professional contacts (*n* = 1,628) was lower than that for private contacts (*n* = 2,138) (see Additional file 1).

The blood donor cohort has a diverse composition in terms of demographic and socioeconomic characteristics, as shown in Table 1. In summary, this information illustrates the diversity and complexity of the blood donor cohort studied, providing an important basis for further scientific analyses. For a comprehensive overview of all features, we refer to the detailed description of the baseline cohort of the SeMaCo study [15].

**Table 1:** Description of the SeMaCo study sample (*n* = 2,138)

	*n*	(%)
**Gender**		
Male	1,105	(51.7)
Female	1,033	(48.3)
**Age**		
18–30 years	535	(25.0)
31–40 years	370	(17.3)
41–50 years	400	(18.7)
51–60 years	518	(24.2)
61–83 years	315	(14.7)
Split according to active labour market*		
Active on the labour market	1,628	(76.1)
Not active on the labour market	510	(23.9)
Education (according to CASMIN**)		
CASMIN low	42	(42.0)
CASMIN middle	1,298	(60.7)
CASMIN high	791	(37.0)
Still without a high school graduation (no CASMIN category)	7	(0.3)
**Marital status**		
Married, living with spouse; registered civil partnership, cohabiting with partner (same-sex)	1,027	(48.0)
Married, living seperately from spouse; registered civil partnership, living separately from partner (same-sex); registered civil partnership annulled (same-sex)	36	(1.6)
Single, divorced, widowed	1075	(50.3)
**Private weekly contact level before 18 March 2020**		
No regular contacts	221	(10.3)
1 to 5	904	(42.3)
6 to 10	576	(26.9)
More than 10	437	(20.4)
**Professional weekly contact level before 18 March 2020**		
No regular contacts	186	(8.7)
1 to 5	453	(21.2)
6 to 10	448	(21.0)
More than 10	842	(39.4)
Not applicable	209	(9.8)
**Private contacts after 18 March 2020**		
Reduction	1,687	(78.9)
No reduction	451	(21.1)
**Professional contacts after 18 March 2020**		
Reduction	1,163	(54.4)
No reduction	975	(45.6)

*Active on the labour market: Fulltime (including professional in-job training or self-employment); part-time (including vocational training,* partial retirement or self-employment); temporarily reduced hours; marginally or intermittently employed; employed occasionally or irregularly advanced training program; federal voluntary service or in voluntary social year; retraining; partial retirement; not active on the labour market: Retired*,* pensioner or in early retirement; school pupil; student; work as a homemaker; caregiver for children or dependent persons*,* not employed; permanently or temporarily unable to work; registered unemployed or looking for work; maternity leave*,* parental leave or other leave of absence*

** CASMIN: Comparative Analyses of Social Mobility in Industrial Nations [17]

### Private and professional contacts by sociodemographic characteristics and by serological result (in combination with vaccination status)

**Fig. 1:**
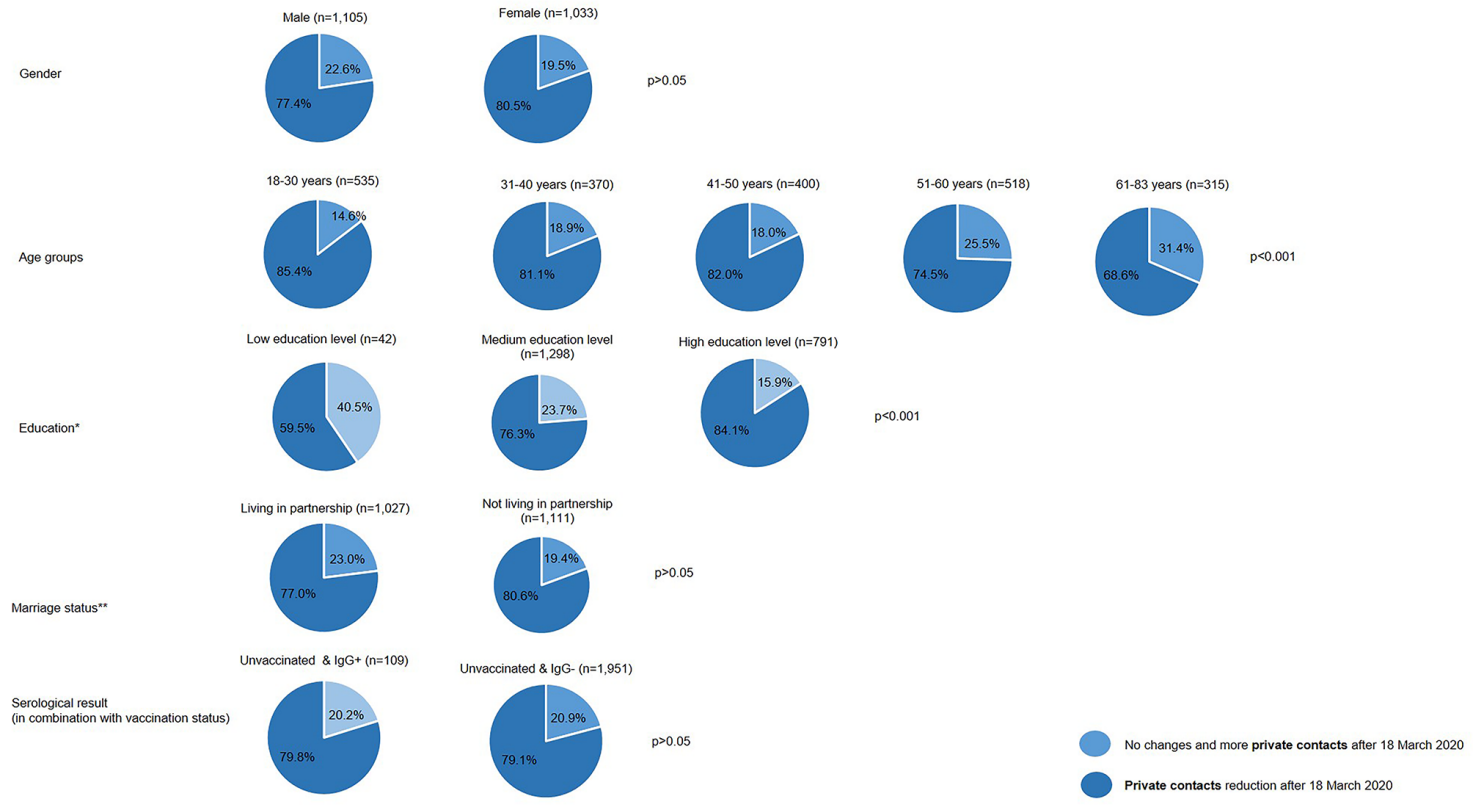
Private contacts by sociodemographic characteristics and serological result (in combination with vaccination status). *Low education = CASMIN 1, medium education = CASMIN 2, high education = CASMIN 3. **Living in a partnership: Married, living with spouse; registered civil partnership, cohabiting with partner (same-sex); Not living in a partnership: Single, divorced, widowed; married, living separately from spouse; registered civil partnership, living separately from partner (same-sex); registered civil partnership annulled (same-sex)

**Fig. 2:**
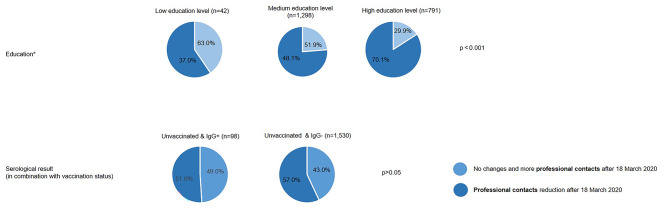
Professional contacts by education and by serological result (in combination with vaccination status). *Low education = CASMIN 1, medium education = CASMIN 2, high education = CASMIN 3

Figures 1 and 2 illustrate significant changes in private and professional contacts within the cohort after March 18, 2020, based on sociodemographic characteristics and serological results. Individuals with low educational attainment reported a 59.5% reduction in private contacts, while 63.0% noted changes or stability in professional contacts. Those with higher education levels showed a greater emphasis on reducing private contacts (84.1%), as well as in the professional setting (70.1%). The gender-specific data on private contact reductions indicate no significant difference in distribution between women (80.5%) and men (77.4%). Similarly, there were no differences in contact reductions between blood donors living in partnerships and those not in partnerships (77.4% vs. 80.5%).

The breakdown of private contacts by age reveals that younger individuals in the blood donor cohort aged 18–30 years were more likely to reduce their private contacts (85.4%) compared to older age groups. Individuals aged 61–83 years (68.6%) exhibited the smallest reduction in their private contacts compared to other age groups.

In order to identify potential COVID-19 infections, participants were divided based on their vaccination status and serological findings (IgG^+^/IgG^−^). In Figures 1 and 2, unvaccinated participants and IgG + individuals were classified by reductions in private and professional contacts at the time of the survey. The group of IgG-positive blood donors reported increased reductions in private contacts since March 18, 2020 (79.8%). In the much larger group of 1,530 unvaccinated individuals with IgG- status, the distribution is almost identical at 79.1%. Regarding professional contacts, 51% of unvaccinated individuals with IgG + status (*n* = 98) reported reductions. There was no significant difference (*p* < 0.05) between unvaccinated individuals with IgG + and IgG- status regarding private and professional contacts.

The results of the regression model are shown in the supplementary file, Table S1.

## Discussion

In this study, we examined changes in private and professional contact behaviours in a blood donor cohort from Magdeburg and its surrounding areas after 18 March, 2020. In the analysed cohort, a general reduction in both private and professional contacts has been observed since the beginning of the pandemic in March 2020. We found that the data revealed differences in contact reductions based on education levels. Individuals with low levels of education reduced their private contacts less, whereas those with high levels of education were more attentive to reducing their contacts. Similar patterns were observed with regard to professional contacts. Individuals with high levels of education had a significantly higher likelihood of reducing their professional contacts compared to those with low levels of education.

In the SeMaCo cohort, there is a balanced gender distribution (male 51.7% vs. female 48.3%) and similar to the gender distribution of the Magdeburg population from 2023 (male 49.4% vs. female 50.6%) [15, 18]. The same can be observed for the average age between the SeMaCo study and the Magdeburg population (43.65 years vs. 45.27 years) [15, 18]. Although the gender and age structure is similar, it is important to note that a blood donor cohort cannot be fully representative of the general population. The differences between blood donors and the general population can be attributed to the “healthy donor effect,” [19] which states that blood donors are generally healthier than the general population and may adhere more closely to health recommendations [20–22]. Therefore, it can be assumed that the blood donor cohorts analyzed here may have been more inclined to implement NPIs during the pandemic compared to the general population.

Existing health disparities in infectious diseases have been well-documented [23–25]. In the early phase of the pandemic (2020), some international studies from the UK and the USA confirmed an increased risk of infection and more severe outcomes of COVID-19 among socioeconomically disadvantaged individuals [26]. Although some studies also showed reverse associations [26–28], a dynamic emerged during the later stages of the pandemic, indicating an elevated risk of infection in socioeconomically disadvantaged groups, both in Germany [29] and internationally [30]. In their study from May to July 2020, Waldhauer et al. (2022) also reported a higher prevalence of contact reductions with increasing educational and occupational status [11]. Our investigation confirmed this observation. Nevertheless, within our examined blood donor cohort, despite a notably higher level of education (CASMIN) there was a simultaneously greater overall restriction of private contacts compared to professional contacts.

After the first wave of the COVID-19 pandemic in spring 2020, it was already clear that the risk of severe COVID-19 outcomes increased with age [31]. Older adults are often affected by age-related comorbidities such as cardiovascular diseases, diabetes mellitus, and lung conditions, which can negatively influence the course of COVID-19. These associations can be confirmed in Germany during the survey period presented here through hospitalization rates from March 2021. According to data from the Intensive Care Register [32], the prevalence of patients requiring intensive care significantly increased from the age group of 50 years and above.

In line with the protective measures communicated by policymakers, especially targeting older adults and individuals with pre-existing conditions [33], it is surprising that our results show an increasing reduction in contact frequency with decreasing age and particularly among retirement-age individuals (61–83 years), who exhibited the lowest reduction in their private contacts compared to other age groups (68.6%). This should be considered in light of the likelihood that younger individuals had higher contact frequencies before the pandemic compared to older individuals. However, similar results can be observed in findings from other studies. During the initial phase of the first COVID-19 outbreak in Portugal, Pasion et al. (2020) found that older adults showed less engagement in protective behaviours with increasing age. The study also showed that older adults perceived the risk to be lower compared to middle-aged adults [34]. However, it is often assumed that older age is associated with higher frequency of preventive practices as well as higher levels of health literacy and concern about the coronavirus [35–37].

### Limitations

The SeMaCo study exclusively includes blood donors from Magdeburg and its surroundings, the results should not be extrapolated on the general population. Blood donors represent only a portion of the adult population, e.g. excluding individuals with comorbidities. The homogeneity of the SeMaCo cohort is reflected, in part, by the predominance of medium and high levels ofeducation, which complicates the interpretation of results regarding variables related to education. While we collected data on education level and type of employment, information on professional status and specific work sectors was lacking. This constitutes a significant limitation, especially in interpreting professional contact frequencies, as no socioeconomic inferences can be made.

It would have been essential to draw conclusions regarding potential COVID-19 infections based on private and/or professional contact reductions. Unfortunately, within our study, this was limited due to the very few unvaccinated and seropositive for SARS-CoV-2 study participants at the time of the survey.

The questions about contact reduction addressed the period before and after 18 March, 2020, and were therefore backward-looking, covering a period of 10 to 13 months. This may have led to bias in assessing contact reductions, as participants were asked to recall and report on both time points in the same survey. Furthermore, our data might also be influenced by desirability bias, as the study was conducted at an early stage of the pandemic when acceptance of containment measures was high. The same applies to the problem of social desirability, where some groups may feel more pressure to present themselves in a socially favourable way on the survey.

### Electronic supplementary material

Below is the link to the electronic supplementary material.

Supplementary Material 1

Supplementary Material 2

## Data Availability

This is an open access article distributed in accordance with the Creative Commons Attribution Non Commercial (CC BY-NC 4.0) license, which permits others to distribute, remix, adapt, build upon this work non-commercially, and license their derivative works on different terms, provided the original work is properly cited, appropriate credit is given, any changes made indicated, and the use is non-commercial. See: http://creativecommons.org/licenses/by-nc/4.0/.The datasets used and/or analyzed during the current study are available from the corresponding author upon reasonable request.

## Abbreviations

BAU: Binding antibody unit
CASMIN: Comparative Analyses of Social Mobility in Industrial Nations
NPI: Non-pharmaceutical interventions
OR: Odds ratio
RKI: Robert Koch Institute
STROBE: Strengthening the Reporting of Observational studies in Epidemiology
