# Supplementary figures and images for: Sociodemographic influences on private and professional contact behaviour during the COVID-19 pandemic in Germany: cross-sectional analysis based on a Regional Blood Donor Cohort
Source: BMC Res Notes. 2024 Jul 27;17:206. doi: 10.1186/s13104-024-06867-9 (PMC11283687; doi:10.1186/s13104-024-06867-9)

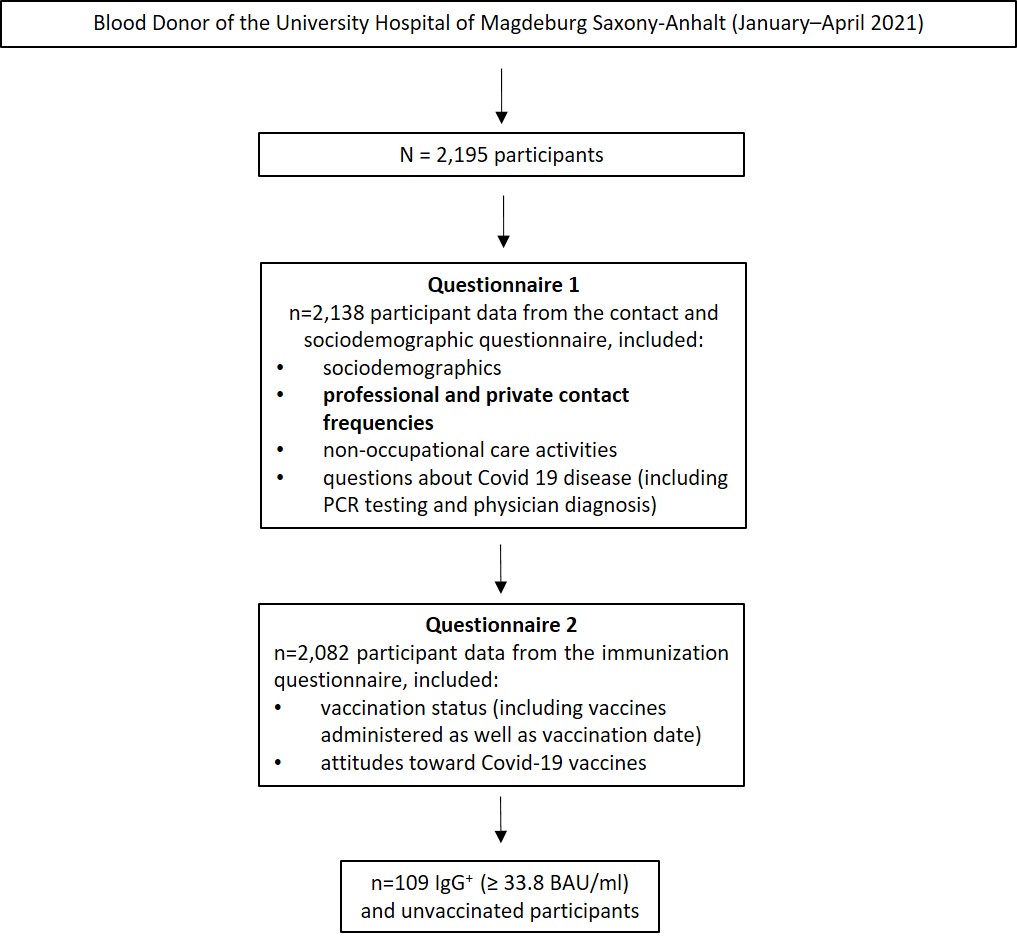

Supplement: Supplementary file 1 — Supplementary Material 1 [file 13104_2024_6867_MOESM1_ESM.jpg]
