# Supplementary material for: Sociodemographic influences on private and professional contact behaviour during the COVID-19 pandemic in Germany: cross-sectional analysis based on a Regional Blood Donor Cohort
Source: BMC Res Notes. 2024 Jul 27;17:206. doi: 10.1186/s13104-024-06867-9 (PMC11283687; doi:10.1186/s13104-024-06867-9)
Supplement: Supplementary file 2 — Supplementary Material 2 [file 13104_2024_6867_MOESM2_ESM.docx]

Table S1: Reduction of private (n=2,138) and professional contacts (n=1,628) after 18 March 2020 by educational status and age group and serological result (in combination with vaccination status), binary logistic regression

| **Reduction of private contacts** | | | |
| --- | --- | --- | --- |
| **Variables** | **Coefficient** | **Odds ratio (95% CI)** | **p-value** |
| **Education** |  |  |  |
| Low Education (ref.) |  |  |  |
| Middle Education | 0.72 | 2.04 (1.07-3.89) | 0.030 |
| High Education | 1.30 | 3.70 (1.91-7.10) | <0.001 |
| **Age** |  | | |
| 18-30 years (ref.) |  |  |  |
| 31-40 years | -0.38 | 0.69 (0.48-0.99) | 0.042 |
| 41-50 years | -0.26 | 0.77 (0.54-1.00) | 0.153 |
| 51-60 years | -0.72 | 0.49 (0.36-0.67) | <0.001 |
| 61-83 years | -1.07 | 0.34 (0.24-0.48) | <0.001 |
| *pseudo-R^2^=0.055* | | | |
| **Reduction of professional contacts** | | | |
| **Variables** | **Coefficient** | **Odds ratio (95% CI)** | **p-value** |
| **Education** |  |  |  |
| Low Education (ref.) |  |  |  |
| Middle Education | 0.45 | 1.57 (0.71-3.47) | 0.262 |
| High Education | 1.38 | 3.99 (1.79-8.85) | <0.001 |
| *pseudo-R^2^=0.066* | | | |
